# Endoscopically-assisted extraction of broken roots or fragments within the mandibular canal: a retrospective case series study

**DOI:** 10.1186/s12903-024-04216-7

**Published:** 2024-04-15

**Authors:** Junqi Jiang, Kenan Chen, Enbo Wang, Denghui Duan, Xiangliang Xu

**Affiliations:** Department of Oral and Maxillofacial Surgery, National Center of Stomatology and National Clinical Research Center for Oral Diseases and National Engineering Research Center of Oral Biomaterials and Digital Medical Devices and Beijing Key Laboratory of Digital Stomatology and Research Center of Engineering and Technology for Computerized Dentistry Ministry of Health and NMPA Key Laboratory for Dental Materials, Peking University School and Hospital of Stomatology, No. 22 Zhongguancun South Avenue, Haidian District, Beijing, 100081 People’s Republic of China

**Keywords:** Endoscope, Fractured roots or fragments, Inferior alveolar nerve, Extraction, Quantitative sensory testing

## Abstract

**Purpose:**

To assess the impact of endoscope-assisted fractured roots or fragments extraction within the mandibular canal, along with quantitative sensory testing (QST) alterations in the inferior alveolar nerve (IAN).

**Methods:**

Six patients with lower lip numbness following mandibular third molar extraction were selected. All patients had broken roots or fragments within the mandibular canal that were extracted under real-time endoscopic assistance. Follow-up assessments were conducted on postoperative days 1, 7, and 35, including a standardized QST of the lower lip skin.

**Results:**

The average surgical duration was 32.5 min, with the IAN exposed in all cases. Two of the patient exhibited complete recovery of lower lip numbness, three experienced symptom improvement, and one patient remained unaffected 35 days after the surgery. Preoperative QST results showed that the mechanical detection and pain thresholds on the affected side were significantly higher than those on the healthy side, but improved significantly by postoperative day 7 in five patients, and returned to baseline in two patients on day 35. There were no significant differences in the remaining QST parameters.

**Conclusions:**

All endoscopic surgical procedures were successfully completed without any additional postoperative complications. There were no cases of deterioration of IAN injury, and lower lip numbness recovered in the majority of cases. Endoscopy allowed direct visualization and examination of the affected nerve, facilitating a comprehensive analysis of the IAN.

## Introduction

The extraction of the mandibular third molar (M3M), a common procedure in oral and maxillofacial surgery, is associated with a high incidence of root fractures. The standard approach is to extract all fractured roots due to the potential risk of infection. However, specific circumstances may permit the conservation of broken roots, for example when they are small, there are no surrounding lesions, or more invasive surgical procedures are required. Factors contributing to root displacement include aggressive clinical procedures, inadequate radiographic examination, and limited visibility [1].

Researchers have proposed several surgical techniques for the extraction of fractured roots, including extended full-thickness flaps, computer-assisted navigation, and endoscopic-assisted approaches [2–4]. The intrusion of fractured roots or crown fragments into the inferior alveolar nerve (IAN) can result in persistent postoperative numbness. Although rare, this can cause significant discomfort in the form of dysesthesia, anesthesia, paresthesia, or hyperalgesia of the skin, mucous membrane, and teeth innervated by IAN. The reported IANI incidence is 0.41-8.10% for temporary injuries and 0.01-3.60% for permanent ones [5]. Even slight damage have the potential to impact the patient’s physical and psychological health [6]. Consequently, dentists often opt to retain and observe fractured roots due to the difficulty in extracting them from the IAN under limited visibility, which could potentially aggravate the nerve damage. Conservative treatment poses certain risks, including persistent numbness, root infection, and root displacement, but these have not been investigated in previous studies.

Endoscopic techniques, serving as a magnifying optical tool, offer operative field magnification and digital recording [7]. Endoscopy has been widely used and reported in oral and maxillofacial surgery. In 2014, Engelke pioneered the use of endoscopy to remove impacted M3Ms without increasing the risk of IAN injury (IANI) [8]. Huang successfully removed residual M3M roots in the maxillofacial space using endoscopy, demonstrating the safety and efficiency of the procedure [9]. Our previous study demonstrated the feasibility of using endoscopy to extract impacted M3Ms adjacent to the IAN and intraoperatively observe IAN exposure [10]. Maxillofacial surgeons have emphasized the importance of adequate IAN visualization during surgical procedures for achieving predictable treatment outcomes and minimizing complications [11].

To evaluate thermal and mechanical somatosensory functions, the German Research Network on Neuropathic Pain has established a standardized Quantitative Sensory Testing (QST) protocol [12]. Yan et al. have validated the sensitivity of QST for detecting abnormalities in IAN function related to somatosensory aspects [13]. The complex situation involving fractured roots penetrating the IAN and the resulting lip numbness presents a challenging scenario with limited effective treatment options [14]. The present study explored the use of endoscopy to remove fractured roots or fragments from the IAN and utilized a standardized QST protocol to record somatosensory functional changes.

## Patients and methods

### Participants

**Fig. 1:**
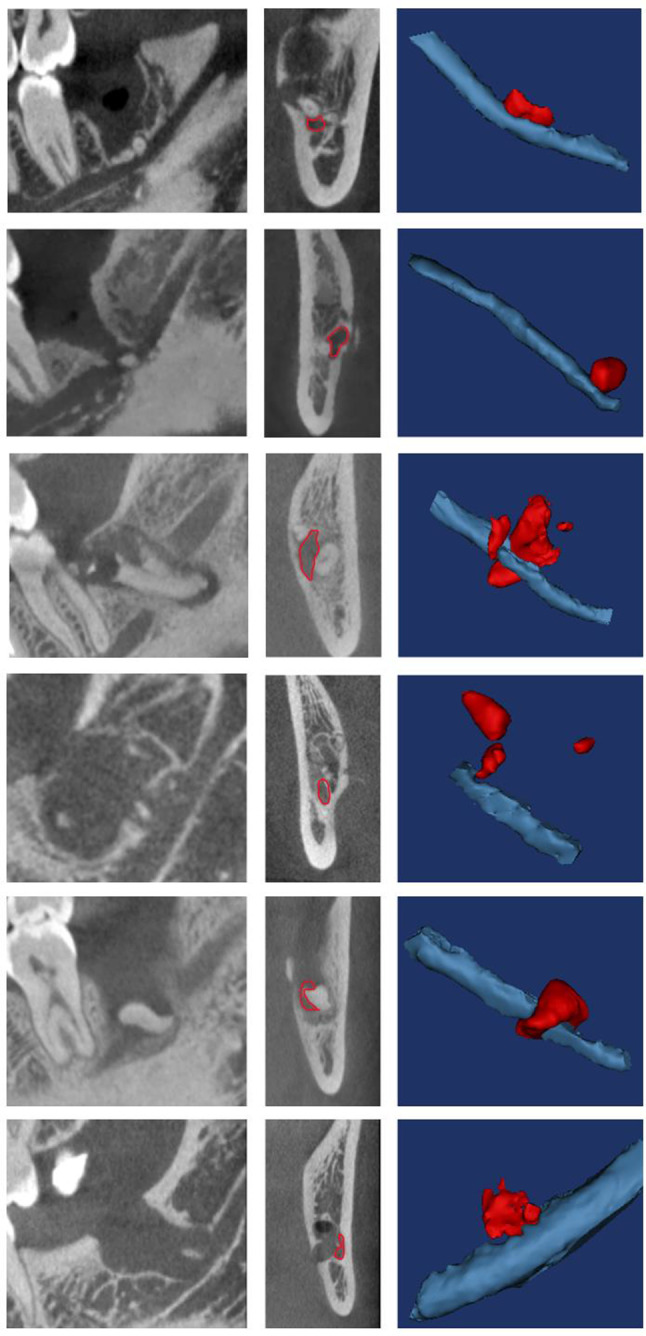
CBCT showing the positional relationship between fractured roots and the mandibular canal (red outline). Mimics software was used to reconstruct the 3D relationship between fractured roots (red) and the IAN (blue)

This study was performed in line with the principles of the Declaration of Helsinki. The biomedical ethics committee of Peking University Hospital of Stomatology approved this study (PKUSSIRB-201949142). All patients requested the removal of fractured roots and signed the informed consent formsand written informed consent was obtained from all participants. Between August 2020 and September 2023, the Department of Oral and Maxillofacial Surgery at Peking University School and Hospital of Stomatology, China, treated six patients with persistent lower lip numbness. The recruitment of patients was consecutive. All patients reported no improvement following the initial surgery, and had no other medical disorders that could affect the results. In five cases, fractured roots entered the IAN, while one case involved bone fragments compressing the IAN. Patient-related information, including age, sex, time between the initial operation, radiographic data, and outcomes, was collected. Cone-beam computed tomography (CBCT) images were acquired using 3D Accuitomo (J Morita Mfg. Corp., Kyoto, Japan), utilizing the following parameters: tube potential of 85–90 kVp, tube current of 5 mA, field of view of 6 cm × 6 cm, and a voxel size of 0.125 mm. The slice thickness and interval were both set at 0.2 mm. The CBCT images were used to determine the location of the residual root, and 3D reconstructions were performed using Mimics software (Mimics research 19.0, Materialise, Belgium) (Fig. 1).

### Endoscopic system

The Storz Hopkins endoscope (Karl Storz, Tuttlingen, Germany; Cat. No. 20223020) and DELON endoscope (Beijing Fanxing Guangdian Medical Treatment Equipment Co., Beijing, China; Cat. No. UHD3840), with a 30° view angle and 4.0-mm diameter, were used in this study. A searching-unit medical endoscope with a cold light type camera was used to record the operation.

### Surgical procedure

**Fig. 2:**
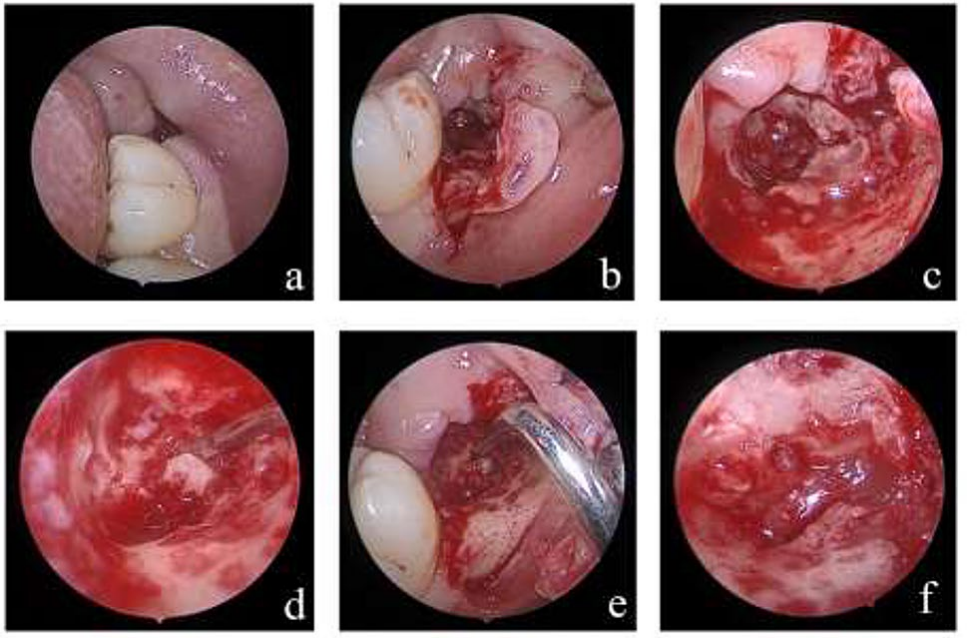
Protocol for fractured root extraction through endoscopic surgery. (**a**) Preoperative images; (**b**) Flap elevation and exposure; (**c**) Scraping of the granulation tissue; (**d**) Removal of the distal root; (**e**) Removal of the mesial root; (**f**) IAN exposure was recorded

All the operations were performed by one surgeon whose level of expertise is the professor. Surgery was performed under local anesthesia (4% articaine with 1:100,000 epinephrine + 2% lidocaine). An angular incision was made on the buccal and distal mucosa of the mandibular second molar and a flap was raised. The endoscope was placed on either the buccal or lingual side, away from the surgical field, to show the entire operative area. All procedures related to residual roots or fragments removing were performed endoscopically. Initially, the granulation tissue was scraped from the socket to identify the roots. Following endoscopic localization of the residual roots or fragments, piezosurgery was used to remove bony hindrances. The residual roots or fragments were removed using microinstruments guided by real-time endoscopic visualization. A final assessment of the IAN was performed and recorded under endoscopic visualization (Fig. 2).

### Clinical variables

The clinical variables were accessed and recorded by one assessor who was aware of the surgery.

The patients indicated their pain levels using a 10-point visual analog scale (VAS) after surgery.

Mouth opening was measured as the distance between the mesio-incisal edges of the upper and lower right central incisors at maximal mouth opening.

Facial swelling was evaluated using horizontal and vertical guides with a flexible tape on four reference points: tragus, outer corner of the mouth, outer canthus of the eye, and the mandibular angle.

$$\begin{aligned} &\text{S}\text{w}\text{e}\text{l}\text{l}\text{i}\text{n}\text{g} \left(\%\right) = (\text{P}\text{o}\text{s}\text{t}\text{o}\text{p}\text{e}\text{r}\text{a}\text{t}\text{i}\text{v}\text{e} \;\text{v}\text{a}\text{l}\text{u}\text{e}-\text{P}\text{r}\text{e}\text{o}\text{p}\text{e}\text{r}\text{a}\text{t}\text{i}\text{v}\text{e} \;\text{v}\text{a}\text{l}\text{u}\text{e})\\ &\quad/\text{P}\text{r}\text{e}\text{o}\text{p}\text{e}\text{r}\text{a}\text{t}\text{i}\text{v}\text{e}\;\text{v}\text{a}\text{l}\text{u}\text{e} \times 100\%;\end{aligned}$$

### Quantitative sensory testing

QST of the IAN involved four assessments on the skin over the mental foramina, both on the operative and contralateral sides. These evaluations were performed 1 week before surgery and 1, 7, and 35 days after the surgery in a quiet room at 21–23 °C by one assessor who was not aware of the surgery. The QST protocol comprised seven subtests, including 13 thermal and mechanical parameters. The parameters included cold detection threshold (CDT), warm detection threshold (WDT), thermal sensory limen (TSL), paradoxical heat sensation (PHS), cold pain threshold (CPT), heat pain threshold (HPT), mechanical detection threshold (MDT), mechanical pain threshold (MPT), dynamic mechanical allodynia (DMA), mechanical pain sensitivity (MPS), wind-up ratio (MUR), vibration detection threshold (VDT), and pressure pain threshold (PPT). The approach described by Yan et al. [13] was utilized for the assessments.

## Results

Table 1 presents the clinical data for the six patients, including four females and two males, with a mean age of 32 years (range: 25–47 years). The interval between the two surgeries ranged from 3 weeks to 1 year. The average second operation time was 32.5 min (range 20–44 min). Lower lip numbness recovered completely in two patients, partially improved in three patients, and remained unchanged in one patient. IAN was exposed in all patients during surgery, and no other postoperative complications occurred. Table 2 outlines the clinical evaluation variables for the six cases. The variables returned to normal 35 days after the operation.

**Table 1:** Clinical data

Case	Gender	Age, years	Time between the first operation	Operation time, min	Recovery of lower lip numbness	IAN exposure	Complications
1	Female	25	3 weeks	20	Complete recovery	Yes	No
2	Female	47	2 months	33	Partial improve	Yes	No
3	Female	26	5 weeks	38	Partial improve	Yes	No
4	Female	35	6 months	40	Partial improve	Yes	No
5	Male	34	1 years	44	Unchanged	Yes	No
6	Male	26	3 weeks	20	Complete recovery	Yes	No

**Table 2:** Clinical evaluation variables

Case	Evaluation variables	Pre	Pos 1	Pos 7	Pos 35
1	Mouth Opening (mm)	35	21	18	32
	VAS	/	2	0.5	0
	Swelling rate (%)	/	-0.49	-0.49	-3.9
2	Mouth Opening (mm)	40	18	38	39
	VAS	/	2.5	0.5	0
	Swelling rate (%)	/	0.88	3.52	0.44
3	Mouth Opening (mm)	47	21	38	43
	VAS	/	0	0	0
	Swelling rate (%)	/	2.04	1.63	0.82
4	Mouth Opening (mm)	51	39	48	54
	VAS	/	2	1	0
	Swelling rate (%)	/	4.58	2.92	0
5	Mouth Opening (mm)	39	32	38	38
	VAS	/	2.5	0	0
	Swelling rate (%)	/	1.87	2.8	1.4
6	Mouth Opening (mm)	41	20	29	42
	VAS	/	2	1.5	0
	Swelling rate (%)	/	6.03	-0.86	-3.02

**Fig. 3:**
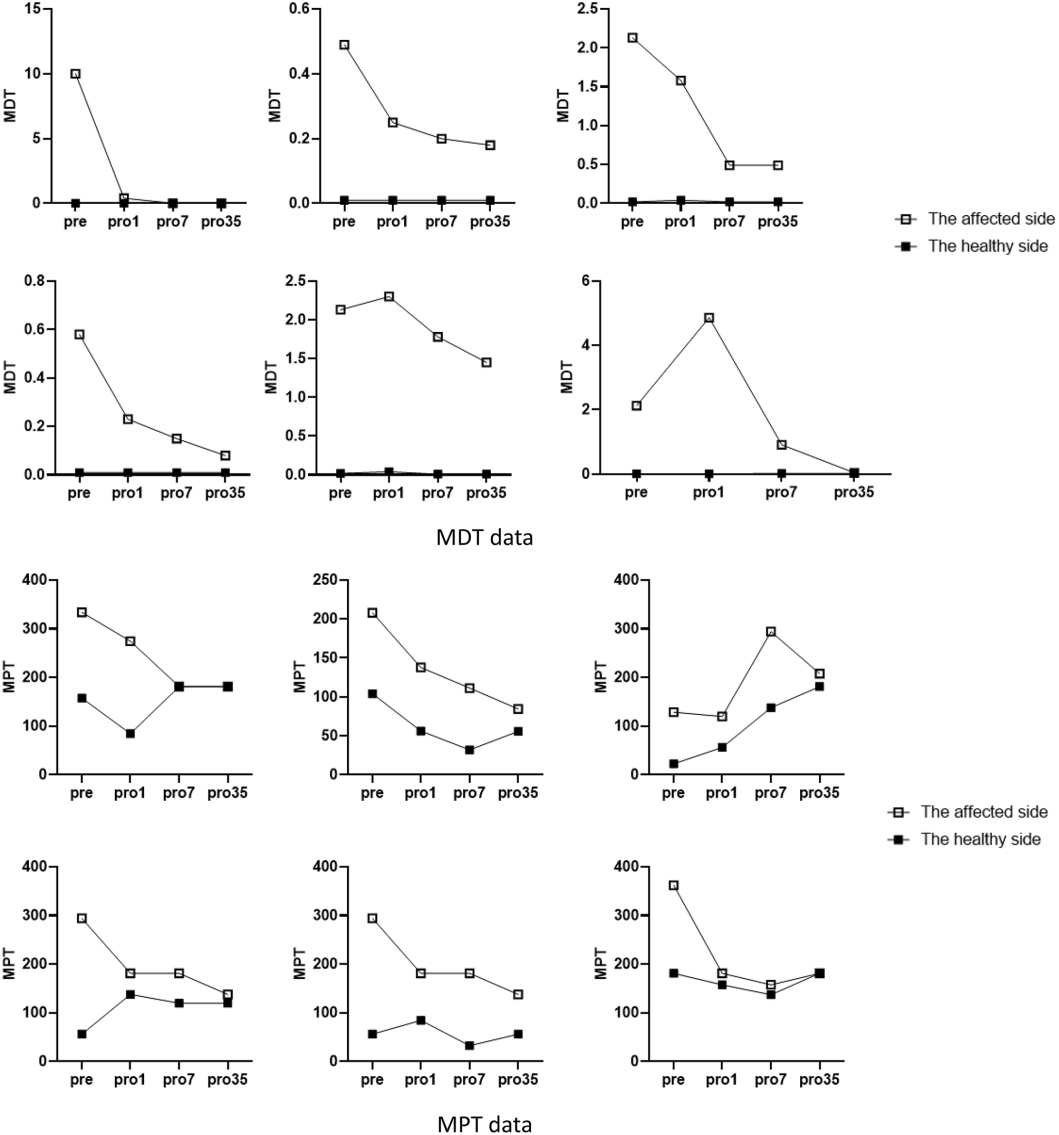
Variations in MDT and MPT data for the six cases. *MDT* mechanical detection threshold, *MPT* mechanical pain threshold

The QST data exhibited minimal disparity before surgery, except for the MDT and MPT. Figure 3 provides the variations in MDT and MPT data for the six cases, demonstrating a significant improvement in five patients by postoperative day 7. Additionally, the affected side of two patients returned to the baseline health status by postoperative day 35.

## Discussion

The proximity of M3M roots to the IAN poses a risk of IANI, leading to lower lip numbness as a postoperative complication of tooth extraction. Even minor sensory changes resulting from IANI can affect the physical and psychological health of the patient [15]. Once fractured roots enter the IAN and the patient experiences persistent lip numbness, the impact of root removal on paresthesia is uncertain. Our study demonstrated that endoscopic removal of fractured M3M roots within the IAN can reduce lip numbness to varying degrees.

Operative visibility in the M3M region is typically poor due to oral cavity restrictions. The field of view is further reduced in case of fractured roots. Retrieval surgery becomes more challenging because of the increased difficulty and risk. Aznar-Arasa et al. suggested that removal might be unnecessary for small root fragments (< 5 mm) or in cases with a high risk of lingual nerve and IANI [16]. Anand et al. proposed leaving root fragments in the absence of associated symptoms or complications [17]. While observation and follow-up are often recommended, the rate of recovery of lower lip numbness remains unknown. There was no research on removing the fractured roots from IAN to relieve lower lip numbness till now. Current studies have mainly focused on the retrieval of fractured roots displaced into the maxillofacial region, relying on CT scans, computer-assisted navigation systems, or surgeon experience for root localization [2–4, 18]. Patient positional changes and poor vision can increase operative difficulty. Compared with the conventional techniques, endoscopy could provide real-time clear operation field and a brighter light source during the surgery. Endoscopy, widely applied in maxillofacial surgery [5–8], was used in our previous study to accurately record IAN exposure and reduce the incidence of IANI after extraction [10]. In the present study, endoscopy was used to enhance real-time visualization of the surgical field, facilitating easier identification of roots and the IAN. Real-time endoscopic assistance, coupled with microscopic instruments, enabled effective extraction of broken roots within the IAN. The endoscopy, as a magnifying optical tool, provided more adequate and direct insight for those complex cases with difficult access than common magnifying glasses. The endoscopy with fine lens tips and different angles could facilitate improved visualization of deep tissue conditions. Meanwhile, endoscopy could record real-time images during the surgery, which could not be achieved by common magnifying glasses and loupes.

Accidental displacement of fractured roots is a rare but serious complication. The timing of removal of such roots remains a subject of debate [18]. Some studies have suggested early removal to reduce complications [1, 9, 18], while others have proposed delayed removal to promote fibrosis and root stabilization [17]. Our results indicate that when fractured roots enter the IAN, earlier removal correlates with better alleviation of lower lip numbness. The patient with the least favorable recovery underwent surgery after a 1-year interval, while those with complete recovery underwent surgery after 3 weeks. None of the patients experienced an increase in pain, swelling, or trismus postoperatively, indicating that endoscopy-assisted techniques did not increase surgical trauma. This finding highlights the minimal invasiveness of endoscopy-assisted techniques without compromising patient comfort.

All patients in our study presented with lower lip numbness following tooth extraction, raising suspicion of IAN compression by broken roots or fragments. A few studies have recommended surgical decompression as a valuable option for nerve injuries caused by endodontic material leaks within the mandibular canal [19, 20]. Liang et al. reported recovery of IAN function after decompression of large mandibular cystic lesions [21], while endoscopic optic nerve decompression has shown benefits for traumatic optic neuropathy [22]. Thus, nerve decompression is a feasible approach for the management of nerve impairment. Our findings also suggest that extracting fractured roots or fragments effectively alleviates IAN compression, promoting the restoration of IAN function. Direct endoscopic observation of the IAN during surgery holds potential for the administration of neurotrophic medications and fostering further research.

QST is a highly sensitive approach for identifying somatosensory abnormalities, including lower lip numbness [23]. An advantage of QST is its ability to assess lower lip numbness using objective data. Consequently, this protocol has been extensively utilized in the oral-facial region. Despite a limited sample size, our study found low sensitivity for QST parameters, except for MDT and MPT. Thermal detection thresholds remained generally unchanged, indicating normal functioning of A fibers and C fibers [24]. Consistent with a study by Porporatti et al. [25], our results suggested that MDT and MPT were the most sensitive QST parameters for IANI. The MDT test was used to determine the minimal force required for subjects to perceive a gentle, non-painful touch [26]. Notably, one patient who was unable to perceive the largest touch before surgery demonstrated complete recovery during follow-up. Data from the affected side showed significant improvement, approaching baseline data of the healthy side in five patients. Improvements in MDT data indicated that extracting broken roots within the mandibular canal effectively reduces lip numbness. The MPT test, similar to MDT, assessed painful sensations [26]. MPT on the affected side was initially higher than that on the healthy side, but decreased after 35 days postoperatively. This indirectly indicates the minimal invasiveness of our surgical procedure without raising the pain threshold on the affected side. Thus, extracting broken roots may contribute to the recovery of pain thresholds. MDT and MPT could serve as viable methods for assessing neurological recovery, with results from larger samples to be presented in a future report.

The primary constraint of our study was the limited sample size because of the low incidence. However, this study firstly introduced a novel approach to extracting fractured roots or fragments within the mandibular canal. The improvement of lower lip numbness was observed during follow-up. We plan to collect more cases in the future and enhance the clinical application of endoscopy in the treatment of IANI.

## Conclusions

For patients with lower lip numbness following M3M extraction, an endoscope may be used for the extraction of residual broken roots or fragments within the mandibular canal. There were no instances of deterioration of IANI, and the majority of cases exhibited recovery of lower lip numbness. Endoscopy allowed direct visualization and assessment of the affected nerve, thereby facilitating a thorough investigation and analysis of the IAN.

## Data Availability

All data generated or analysed during this study are included in this published article.
